# Association of Coffee and Caffeine Intake With Irritable Bowel Syndrome in Adults

**DOI:** 10.3389/fnut.2021.632469

**Published:** 2021-06-15

**Authors:** Glareh Koochakpoor, Asma Salari-Moghaddam, Ammar Hassanzadeh Keshteli, Ahmad Esmaillzadeh, Peyman Adibi

**Affiliations:** ^1^Maragheh University of Medical Sciences, Maragheh, Iran; ^2^Department of Community Nutrition, School of Nutritional Sciences and Dietetics, Tehran University of Medical Sciences, Tehran, Iran; ^3^Department of Medicine, University of Alberta, Edmonton, AB, Canada; ^4^Obesity and Eating Habits Research Center, Endocrinology and Metabolism Molecular-Cellular Sciences Institute, Tehran University of Medical Sciences, Tehran, Iran; ^5^Department of Community Nutrition, School of Nutrition and Food Science, Isfahan University of Medical Sciences, Isfahan, Iran; ^6^Isfahan Gastroenterology and Hepatology Research Center, Isfahan University of Medical Sciences, Isfahan, Iran

**Keywords:** coffee, caffeine, irritable bowel syndrome, IBS, IBS severity

## Abstract

The aim of this study was to investigate the association between coffee and caffeine intake and odds of IBS and its severity in adult population. In this cross-sectional study, dietary intakes of 3,362 Iranian adults were examined using a validated dish-based 106-item Semi-quantitative Food Frequency Questionnaire (DS-FFQ). Coffee and caffeine intake was assessed using the DS-FFQ. IBS was assessed using a modified Persian version of Rome III questionnaire. After adjustment for potential confounders, we found that individuals who were taking coffee weekly or more had greater odds of IBS (OR:1.44; 95% CI: 1.02-2.04) than those who never drinking coffee. In addition, participants in the top tertile of caffeine intake (≥106.5 mg/d) had 47% greater odds of IBS compared to those in the bottom tertile (<69.4 mg/d) (OR: 1.47; 95% CI: 1.14-1.87). By gender, a significant association was observed between caffeine intake and odds of IBS among women (OR for those in the highest tertile vs. lowest tertile: 1.48; 95% CI: 1.10-2.00), but not in men (OR: 1.47; 95% CI: 0.94-2.30). In addition, a significant positive association was seen between caffeine intake and odds of IBS among subjects with BMI ≥ 25 kg/m^2^ (OR for those in the highest tertile vs. lowest tertile: 1.72; 95% CI: 1.20-2.48). There was a significant association between caffeine intake and IBS severity among subjects with BMI ≥ 25 kg/m^2^ (OR: 1.04; 95% CI: 1.01-2.62). In conclusion, coffee and caffeine consumption was associated with increased odds of IBS in the whole study population. The association between caffeine and odds of IBS was also significantly positive among women and overweight or obese subjects (BMI ≥ 25 kg/m^2^). In addition, we found a significant relationship between caffeine intake and severity of IBS symptoms among overweight or obese subjects (BMI ≥ 25 kg/m^2^).

## Introduction

Irritable Bowel Syndrome (IBS) is a common disorder of the gastrointestinal (GI) tract. In USA, 10-20% of the adult population suffer from IBS (1, 2). It is not directly associated with increased mortality rates, but it is associated with reduced health-related quality of life (3, 4). In addition, reduced productivity and high medical costs imposes a significant burden to both patients and the society (5). Therefore, identification and modification of its contributing factors is of high priority.

Although, the interaction between brain and the gut, disturbance in the gut neuroendocrine, aberrant central nervous system, gut dysmotility, and intestinal microbiota are involved in IBS development (6, 7), it is well-documented that diet along with other lifestyle-related variables are among the main risk factors. Almost 50% of patients with IBS have experienced exacerbation of symptoms after eating (8, 9). In addition, consumption of low fat diet, poorly absorbed carbohydrates, and spicy foods has been associated with reduced IBS symptoms (10, 11).

Coffee is a popular beverage with well-known effects on central nervous system (12), gut microbiome (13), gastrointestinal function, and gut motility (14), however, few studies have examined the association between coffee and caffeine intake and odds of IBS. Bohn et al. (15) investigated the effect of coffee intake on severity of IBS symptoms in a cross-sectional study on 197 IBS patients. They introduced coffee as one of top 10 foods causing dyspepsia, pain, and loose stools in IBS patients. It must be kept in mind that the results of their study cannot be generalized to other societies with different lifestyle habits. In addition, they have examined such an association in patients with IBS, not the whole general population to find the association between coffee intake with IBS *per se*. To our knowledge, there is no study examining the association between coffee and caffeine intake and odds of IBS and its severity in a general population. Given the nutrition transition in developing countries and changing habits in these countries from tea consumption to coffee consumption, assessing the association between coffee and caffeine intake and odds of IBS is particularly relevant in these populations. Therefore, the aim of this study was to investigate the association between coffee and caffeine intake and odds of IBS and its severity in Iranian adult population.

## Methods and Materials

### Participants

The present cross-sectional study was done based on the study on the Epidemiology of Psychological Alimentary Health and Nutrition (SEPAHAN) project. Information about SEPAHAN study has been published elsewhere (16). In summary, this study was performed on Iranian adults working in 50 different healthcare centers affiliated to Isfahan University of Medical Sciences (IUMS) across Isfahan province. Performing in two separate phases between April 2010 and May 2010, 10,087 questionnaires were sent in the first phase to study participants. This questionnaire had question as about demographic information, medical history, anthropometric measures, lifestyle, and dietary intakes. At this phase, 8,691 questionnaires were filled and returned (response rate: 86.16%). Then in the second stage, a bunch of questionnaires about gastrointestinal health was sent and finally 6,239 completed questionnaires returned at this phase (response rate: 64.6%). To analyse data from both phases, we tried to merge data from the first and second phases. After processing data, we had a complete information on 4,763 subjects about diet and functional gastrointestinal disorders. In the present analysis, we excluded persons with non-feasible total daily energy intake (outside the range of 800-4,200 kcal per day). In addition, individuals with missing data on any relevant variables were also excluded. Therefore, data from 3,362 subjects were included in the current analysis. All study subjects had provided informed written consents and the study protocol was ethically approved by the regional committee of bioethics of Isfahan University of Medical Sciences.

### Dietary Intakes Assessment

A self-administered, Willett-format, Dish-based, 106- item Semi-quantitative Food Frequency Questionnaire (DS-FFQ), was used to assess dietary intakes. Detailed information on design, foods included and validity of this questionnaire has been given elsewhere (17). The questionnaire contained 106 food items in five various categories: (1) mixed dishes (29 items); (2) grains (10 items); (3) dairy products (9 items); (4) fruits and vegetables (22 items); and (5) miscellaneous food items and beverages (36 items). The portion size of foods and mixed dishes was given for each item in front of that item in the questionnaire. Frequency response categories for each four item ranged from “never or <1/month” to “≥ 12 times/day.” Finally, daily intake of all foods and dishes was computed considering the given portion size and frequency responses (18).

Comparing information from the DS-FFQ and the average of three detailed dietary records as the gold standard, we found that the questionnaire works well in estimating long-term dietary intakes (17).

### Calculation of Coffee and Caffeine Intake

To assess coffee intake, participants were asked to report the average number of glasses of coffee they usually consume in the preceding year. Frequency response categories for coffee intake in the questionnaire were as follow: “never or <1 glass/month,” “1-3 glasses/month,” “1 glass/week,” “2-4 glasses/week,” “5-6 glasses/week,” “1 glass/day,” “2-3 glasses/day,” “4-5 glasses/day,” and “≥6 glasses of coffee in a day.” In the analysis, we classified people in terms of coffee intake as “none-user,” “monthly user,” or “weekly or more user.” Classification of people based on cups of coffee intake per day was not possible due to low number of people drinking coffee daily. Total caffeine intake was estimated by summing up the caffeine that participants took from all caffeine-containing foods and beverages (types of chocolate, cocoa, tea, soft drink, coffee, and espresso). In terms of caffeine intake, people were categorized based on tertile cut-off points (<69.4, 69.4-106.4, and ≥106.5 mg/d). This is a usual method in nutritional epidemiology for several nutrients due to lack of a specific cut-off point. However, in terms of foods, categorization should be done by a method that is meaningful for public people because of their use in daily dietary recommendations. This is why our approach for categorization of people in terms of coffee consumption was different with caffeine intake. As coffee is not a usual drink in Iran and most people do not consume coffee regularly, we categorized people in terms of coffee intake as none, monthly, weekly or more.

### Assessment of IBS

A modified Persian version of the Rome III questionnaire (19), as part of the main comprehensive questionnaire, was used for assessment of IBS. It must be mentioned that we modified the descriptors in the original questionnaire to only four item rating scale (i.e., never or rarely, sometimes, often, and always). This was done because in our pilot study we found that it was difficult for participants to discriminate the descriptors used in the original version of Rome III questionnaire (never, <1 day/month, 1 day/month, 2-3 days/month, 1 day/week, >1 day/week, and every day). According to the Rome III criteria, IBS was defined as a condition characterized by recurrent abdominal pain or discomfort at least sometimes in the previous 3 months along with two or more of the following criteria: improvement with defecation, pain with a change in stool frequency, and pain with a change in form (appearance) of stool. IBS subtypes were also defined based on ROME III guidelines (19). The severity of IBS was examined by asking participants about the severity of their abdominal pain in the previous 3 months. They were able to choose one of these responses: mild, moderate, severe, or very severe.

### Assessment of Other Variables

We collected data about age, marital status, smoking status, sex, medication use, and disease history (diabetes and colitis) using self-administered questionnaires. Data on anthropometric variables were also collected using a validated self-reported questionnaire. Physical activity was assessed using the General Practice Physical Activity Questionnaire (20), and participants were classified into two categories: physically active (≥1 h/wk) and physically inactive (<1 h/wk). Data on diet-related habits including meal regularity (often or always/never or occasionally), chewing efficiency (a lot/not a lot), breakfast skipping (skipper/non-skipper), and intra-meal fluid intake (<3 glasses/≥3 glasses) were also assessed through the use of a pretested questionnaire. Dental status was also examined and subjects were categorized as “having all teeth,” “lost 1-5 teeth,” and “lost >5 teeth.”

### Statistical Analysis

General characteristics of study participants across categories of coffee (none, monthly, weekly or more) and caffeine intake (<69.4, 69.4-106.4, and ≥106.5 mg/d) were presented as means ± SDs for continuous variables and percentages for categorical variables. To examine the differences across categories, we used ANOVA for continuous variables and chi-square test for categorical variables. We used binary logistic regression to estimate odds ratios (ORs) and 95% confidence intervals (CIs) for the presence of IBS and its subtypes across categories of coffee and caffeine intake in crude and multivariable-adjusted model. The trend of ORs across categories of coffee and caffeine intake was determined by considering categories of coffee and caffeine intake as ordinal variables in the logistic regression analysis. We also used multivariable ordinal logistic regression to estimate ORs and 95% CIs for assessing IBS severity (mild/moderate/severe/very severe) across categories of coffee and caffeine intake in crude and multivariable-adjusted model. In these analyses, supplement use (yes/no) was controlled for in the first model. Dietary fiber intake (continuous) was controlled for in the second model. Age (continuous), sex (male/female), energy intake (continuous), BMI (continuous), physical activity (<1 h/week/≥1 h/week), smoking status (non-smoker/former smokers and current smokers), medication use (yes/no), self-reported diabetes (yes/no) and colitis (yes/no), psychological distress (yes/no), meal regularity (often or always/never or occasionally), chewing sufficiency (a lot/not a lot), intra-meal fluid consumption (<3 glasses/≥3 glasses), breakfast skipping (skipper/non-skipper), dental status (have all teeth/lost 1-5 teeth/lost >5 teeth), and dietary fiber intake (continuous) were adjusted for in the multivariable model. All statistical analyses were done using the Statistical Package for Social Sciences (version 20; SPSS Inc.). *P* < 0.05 was considered as statistically significant.

## Results

Mean total caffeine intake among whole study participants was 99.10 mg/d. Mean caffeine intake across its tertiles was as follow: 29.96 mg, 74.8 mg, and 191.8 mg/d. General characteristics of study participants across categories of coffee and caffeine intake are shown in Table 1. Participants who consumed coffee weekly or more were more likely to be physically active and current smoker, use dietary supplements, and had higher energy intakes and less likely to lose their teeth compared with those who did not consume coffee. In terms of caffeine intake, those in the top tertile of caffeine intake were more likely to be older, current smoker, had regular meal pattern, and had higher energy intakes and less likely to be female and lose their teeth compared with those in the bottom tertile. No other significant differences were found in terms of other variables.

**Table 1:** General characteristics of study participants across categories of coffee and caffeine intake^a^.

	**Coffee intake**				**Caffeine intake**			
	**None** ** (*n* = 2308)**	**Monthly** ** (*n* = 667)**	**Weekly or more** ** (*n* = 387)**	***P*-value^**b**^**	**T_**1**_** ** (*n* = 1107)**	**T_**2**_** ** (*n* = 1133)**	**T_**3**_** ** (*n* = 1122)**	***P*-value^**b**^**
Age (y)	36.02 ± 7.6	36.51 ± 8.3	35.5 ± 7.2	0.26	35.7 ± 8.1	35.9 ± 7.7	37.1 ± 7.6	<0.001
BMI (kg/m^2^)	24.87 ± 3.8	24.59 ± 3.7	24.75 ± 3.4	0.32	24.8 ± 3.9	24.8 ± 3.7	25.07 ± 3.7	0.21
Energy (kcal/d)	2302.9 ± 815	2545.5 ± 771	2852 ± 725	<0.001	2148.1 ± 807	2375.9 ± 788	2609.3 ± 817	<0.001
Female (%)	58.8	57.6	64.8	0.11	60.4	60.6	53.7	0.001
Married (%)	82	78.7	79.2	0.19	80.7	81.2	83.1	0.65
Physically active (≥1 h/week) (%)	12	16.9	12.5	0.01	13.6	11.3	14.7	0.05
Current smokers (%)	12.2	15.3	16.7	0.02	12.7	12.5	16.1	0.02
Disease history (%)	3.2	1.4	2.8	0.10	3.6	2.6	2.7	0.31
Medication use (%)	6	5.1	7	0.54	6.6	5.6	6.1	0.60
Supplement use(%)	28.4	32.8	39	<0.001	31.3	29.3	29.4	0.49
Regular meal pattern (%)				0.32				0.01
Often or always	61.7	61.3	57.1		57	62.8	61.1	
Never or occasionally	38.3	38.7	42.9		43	37.2	38.9	
Chewing sufficiency (%)				0.77				0.39
A lot	13	11.8	12.7		13.4	13.7	11.9	
Not a lot	87	88.2	87.3		86.6	86.3	88.1	
Fluid consumption (%)				0.31				0.18
<3 glasses	3	4.2	2.5		4	2.6	3.1	
≥3 glasses	97	95.8	97.5		96	97.4	96.9	
Breakfast skipping (Skippers) (%)	7.3	5.7	9.8	0.11	7.5	7	7.5	0.88
Tooth loss (%)				<0.001				0.01
Have all	32.9	34.7	49.5		35.9	31.8	33.1	
Lost 1-5 teeth	59.5	38.5	46.6		57	61.5	57.3	
Lost > 5 teeth	7.6	6.8	4		7.1	6.6	9.6	
Psychological distress (%)	23.3	22.4	24.2	0.84	22.2	23.3	23.9	0.62

a *All values are mean ± SD, unless indicated;*

b *ANOVA for continuous variables and chi-squared test for categorical variables*.

Crude and multivariable-adjusted ORs and 95% CIs for IBS across categories of coffee and caffeine intake are presented in Table 2. In the whole study population, those who consumed coffee weekly or more had higher odds of IBS compared to those who did not take coffee (OR: 1.44; 95% CI: 1.02-2.04). By sex, there was no significant association between coffee consumption and odds of IBS in either gender (for men: OR: 1.44; 95% CI: 0.75-2.76 and for women: OR: 1.45; 95% CI: 0.96-2.19). By BMI, we failed to find any significant association between coffee consumption and odds of IBS in normal weight (BMI <25 kg/m^2^) and overweight or obese (BMI ≥ 25 kg/m^2^) individuals either before or after controlling for potential confounders.

**Table 2:** Crude and multivariable-adjusted ORs and 95% CIs for IBS across categories of coffee and caffeine intake^a^.

	**Coffee intake**				**Caffeine intake**			
	**None** ** (*n* = 2308)**	**Monthly** ** (*n* = 667)**	**Weekly or more** ** (*n* = 387)**	***P*-trend**	**T_**1**_** ** (*n* = 1107)** ** <69.4 mg/d**	**T_**2**_** ** (*n* = 1133)** ** 69.4-106.4 mg/d**	**T_**3**_** ** (*n* = 1122)** ** ≥106.5 mg/d**	***P*-trend**
**Whole population**								
Crude	1.00	1.14 (0.90-1.43)	1.21 (0.91-1.61)	0.10	1.00	1.18 (0.96-1.45)	1.32 (1.08-1.61)	0.006
Model I	1.00	1.21 (0.89-1.41)	1.16 (0.87-1.54)	0.19	1.00	1.19 (0.97-1.46)	1.33 (1.09-1.63)	0.005
Model II	1.00	1.18 (0.93-1.48)	1.26 (0.95-1.68)	0.04	1.00	1.22 (0.99-1.50)	1.39 (1.14-1.71)	0.001
Model III^b^	1.00	1.34 (1.02-1.76)	1.44 (1.02-2.04)	0.007	1.00	1.28 (1.01-1.63)	1.47 (1.14-1.87)	0.002
**Male**								
Crude	1.00	0.88 (0.59-1.31)	1.17 (0.71-1.94)	0.81	1.00	1.17 (0.83-1.66)	1.23 (0.88-1.72)	0.21
Model I	1.00	0.86 (0.58-1.28)	1.12 (0.68-1.86)	0.97	1.00	1.19 (0.84-1.68)	1.24 (0.89-1.73)	0.20
Model II	1.00	0.90 (0.60-1.34)	1.22 (0.74-2.02)	0.68	1.00	1.24 (0.87-1.75)	1.32 (0.94-1.85)	0.10
Model III^c^	1.00	1.03 (0.64-1.67)	1.44 (0.75-2.76)	0.34	1.00	1.45 (0.92-2.26)	1.47 (0.94-2.30)	0.10
**Female**								
Crude	1.00	1.32 (0.99-1.76)	1.20 (0.85-1.70)	0.08	1.00	1.18 (0.92-1.53)	1.44 (1.11-1.86)	0.005
Model I	1.00	1.31 (0.98-1.74)	1.17 (0.83-1.66)	0.12	1.00	1.19 (0.92-1.54)	1.44 (1.11-1.85)	0.005
Model II	1.00	1.38 (1.03-1.84)	1.25 (0.88-1.77)	0.04	1.00	1.21 (0.94-1.56)	1.51 (1.16-1.95)	0.002
Model III^c^	1.00	1.54 (1.10-2.14)	1.45 (0.96-2.19)	0.01	1.00	1.22 (0.91-1.62)	1.48 (1.10-2.00)	0.009
**BMI** **<** **25 (kg/m**^**2**^**)**								
Crude	1.00	0.99 (0.72-1.37)	1.12 (0.76-1.64)	0.61	1.00	1.08 (0.81-1.42)	1.10 (0.83-1.46)	0.47
Model I	1.00	0.98 (0.71-1.35)	1.05 (0.72-1.55)	0.83	1.00	1.10 (0.83-1.45)	1.14 (0.86-1.52)	0.34
Model II	1.00	1.04 (0.75-1.43)	1.18 (0.80-1.74)	0.40	1.00	1.11 (0.84-1.47)	1.18 (0.88-1.57)	0.25
Model III^d^	1.00	1.29 (0.90-1.86)	1.43 (0.91-2.24)	0.05	1.00	1.18 (0.86-1.63)	1.26 (0.90-1.77)	0.16
**BMI** **≥** **25 (kg/m**^**2**^**)**								
Crude	1.00	1.33 (0.94-1.87)	1.23 (0.79-1.93)	0.12	1.00	1.39 (1.02-1.90)	1.57 (1.16-2.13)	0.003
Model I	1.00	1.30 (0.92-1.83)	1.20 (0.77-1.89)	0.17	1.00	1.39 (1.02-1.90)	1.56 (1.15-2.11)	0.004
Model II	1.00	1.36 (0.96-1.92)	1.27 (0.81-1.99)	0.09	1.00	1.47 (1.07-2.01)	1.67 (1.23-2.27)	0.001
Model III^d^	1.00	1.50 (1.00-2.24)	1.50 (0.86-2.60)	0.03	1.00	1.39 (0.96-2.02)	1.72 (1.20-2.48)	0.003

a *Values are OR (95% CIs)*.

*Model I: adjusted for supplement use*.

*Model II: adjusted for dietary fiber intake*.

b *Model III: adjusted for age, sex, energy, BMI, physical activity, smoking status, medication use, disease history (diabetes, colitis), psychological distress, regular meal pattern, chewing sufficiency, fluid consumption, breakfast skipping, dental status, and dietary fiber intake*.

c *Model III: adjusted for age, energy, BMI, physical activity, smoking status, medication use, disease history (diabetes, colitis), psychological distress, regular meal pattern, chewing sufficiency, fluid consumption, breakfast skipping, dental status, and dietary fiber intake*.

d *Model III: adjusted for age, sex, energy, physical activity, smoking status, medication use, disease history (diabetes, colitis), psychological distress, regular meal pattern, chewing sufficiency, fluid consumption, breakfast skipping, dental status, and dietary fiber intake*.

Individuals in top tertile of caffeine intake had greater odds of IBS compared with those in the bottom tertile (OR: 1.47; 95% CI: 1.14-1.87). When we did the analyses by sex, we did not observe any significant association between caffeine intake and odds of IBS among men (OR: 1.47; 95% CI: 0.94-2.30); however, a significant positive association was seen among women (OR: 1.48; 95% CI: 1.10-2.00). By BMI, overweight or obese subjects (BMI ≥ 25 kg/m^2^) in the highest tertile of caffeine intake were 72% more likely to have IBS compared with those in the lowest tertile (OR: 1.72; 95% CI: 1.20-2.48). However, no significant association was found among normal weight subjects (BMI <25 kg/m^2^).

Crude and multivariable-adjusted ORs and 95% CIs for IBS severity across different categories of coffee and caffeine intake are presented in Table 3. There was no significant association between coffee consumption and odds of IBS severity in the whole population or when we analyzed data stratified by gender or BMI status. We found a significant positive association between caffeine intake and IBS severity in overweight or obese (BMI ≥ 25 kg/m^2^) individuals (OR: 1.04; 95% CI: 1.01-2.62). We also did not observe any significant association between caffeine intake and odds of IBS severity in the whole population or in the stratified analysis by gender.

**Table 3:** Crude and multivariable-adjusted ORs and 95% CIs for IBS severity across categories of coffee and caffeine intake^a^.

	**Coffee intake**			**Caffeine intake**		
	**None** ** (*****n*** **=** **2308)**	**Monthly** ** (*****n*** **=** **667)**	**Weekly or more ** **(*****n*** **=** **387)**	**T**_**1**_ **** ** (*****n*** **=** **1107)** **** ** <69.4 mg/d**	**T**_**2**_**** ** (*****n*** **=** **1133)** ** 69.4-106.4 mg/d**	**T**_**3**_**** ** (*****n*** **=** **1122)** **** **≥106.5 mg/d**
**Whole population**						
Crude	1.00	1.32 (0.60-1.15)	2.06 (0.72-1.58)	1.00	2.69 (0.76-1.31)	2.11 (0.79-1.36)
Model I	1.00	1.36 (0.61-1.16)	2.06 (0.72-1.58)	1.00	2.60 (0.75-1.30)	2.17 (0.79-1.35)
Model II	1.00	1.40 (0.61-1.18)	1.88 (0.74-1.63)	1.00	2.51 (0.77-1.33)	1.73 (0.82-1.42)
Model III^b^	1.00	1.32 (0.60-1.15)	2.06 (0.72-1.58)	1.00	2.69 (0.76-1.31)	2.11 (0.79-1.36)
**Male**						
Crude	1.00	1.49 (0.44-1.38)	1.51 (0.66-2.69)	1.00	2.13 (0.56-1.51)	1.84 (0.70-1.80)
Model I	1.00	1.49 (0.44-1.38)	1.51 (0.66-2.69)	1.00	2.24 (0.57-1.53)	1.78 (0.71-1.82)
Model II	1.00	1.53 (0.45-1.40)	1.46 (0.67-3.02)	1.00	2.46 (0.59-1.59)	1.57 (0.74-1.92)
Model III^c^	1.00	1.12 (0.27-1.16)	1.77 (0.53-3.10)	1.00	1.15 (0.32-1.18)	1.39 (0.38-1.37)
**Female**						
Crude	1.00	1.50 (0.58-1.28)	2.36 (0.59-1.54)	1.00	2.15 (0.75-1.45)	2.43 (0.73-1.42)
Model I	1.00	1.70 (0.59-1.30)	2.40 (0.59-1.54)	1.00	2.31 (0.74-1.43)	2.48 (0.73-1.41)
Model II	1.00	1.72 (0.59-1.31)	2.59 (0.61-1.58)	1.00	2.13 (0.75-1.46)	2.11 (0.75-1.47)
Model III^c^	1.00	2.59 (0.65-1.57)	2.52 (0.56-1.69)	1.00	1.44 (0.82-1.70)	1.60 (0.78-1.67)
**BMI** **<** **25 (kg/m**^**2**^**)**						
Crude	1.00	1.19 (0.47-1.14)	1.80 (0.48-1.50)	1.00	1.57 (0.59-1.26)	1.58 (0.59-1.26)
Model I	1.00	1.20 (0.48-1.15)	1.82 (0.48-1.51)	1.00	1.51 (0.58-1.24)	1.51 (0.58-1.24)
Model II	1.00	1.22 (0.48-1.16)	1.92 (0.49-1.55)	1.00	1.58 (0.59-1.26)	1.75 (0.61-1.30)
Model III^d^	1.00	1.32 (0.46-1.24)	1.35 (0.36-1.37)	1.00	1.81 (0.58-1.47)	1.11 (0.45-1.08)
**BMI** **≥** **25 (kg/m**^**2**^**)**						
Crude	1.00	2.47 (0.58-1.59)	1.55 (0.70-2.22)	1.00	1.61 (0.77-1.74)	1.29 (0.84-1.87)
Model I	1.00	2.54 (0.59-1.61)	1.56 (0.70-2.22)	1.00	1.61 (0.77-1.74)	1.29 (0.84-1.87)
Model II	1.00	2.69 (0.60-1.64)	1.48 (0.72-2.28)	1.00	1.41 (0.80-1.83)	1.16 (0.89-2.01)
Model III^d^	1.00	2.10 (0.61-1.96)	1.37 (0.71-2.78)	1.00	1.44 (0.76-2.02)	1.04 (1.01-2.62)

a *Values are OR (95% CIs)*.

*Model I: adjusted for supplement use*.

*Model II: adjusted for dietary fiber intake*.

b *Model III: adjusted for age, sex, energy, BMI, physical activity, smoking status, medication use, disease history (diabetes, colitis), psychological distress, regular meal pattern, chewing sufficiency, fluid consumption, breakfast skipping, dental status, and dietary fiber intake*.

c *Model III: adjusted for age, energy, BMI, physical activity, smoking status, medication use, disease history (diabetes, colitis), psychological distress, regular meal pattern, chewing sufficiency, fluid consumption, breakfast skipping, dental status, and dietary fiber intake*.

d *Model III: adjusted for age, sex, energy, physical activity, smoking status, medication use, disease history (diabetes, colitis), psychological distress, regular meal pattern, chewing sufficiency, fluid consumption, breakfast skipping, dental status, and dietary fiber intake*.

Crude and multivariable-adjusted ORs and 95% CIs for IBS subtypes across different categories of coffee and caffeine intake are presented in Table 4. After controlling for potential confounders, we found that those who were taking coffee weekly or more had 66% higher odds of IBS-C compared with those who did not consume (OR: 1.66; 95% CI: 1.00-2.75). In addition, participants in the highest tertile of caffeine intake had higher odds of IBS-C compared with those in the lowest tertile (OR: 1.49; 95% CI: 1.02-2.16). No other overall association was seen between coffee or caffeine consumption and other types of IBS.

**Table 4:** Crude and multivariable-adjusted ORs and 95% CIs for IBS subtypes across categories of coffee and caffeine intake^a^.

	**Coffee intake**				**Caffeine intake**			
	**None** ** (*n* = 2308)**	**Monthly** ** (*n* = 667)**	**Weekly or more** ** (*n* = 387)**	***P*-trend**	**T_**1**_** ** (*n* = 1107)** ** <69.4 mg/d**	**T_**2**_** ** (*n* = 1133)** ** 69.4-106.4 mg/d**	**T_**3**_** ** (*n* = 1122)** ** ≥106.5 mg/d**	***P*-trend**
**IBS-C**									
Crude	1.00	1.54 (1.09-2.15)	1.80 (1.21-2.68)	0.001	1.00	1.11 (0.79-1.55)	1.56 (1.14-2.15)	0.004
Model I	1.00	1.51 (1.08-2.12)	1.73 (1.16-2.59)	0.001	1.00	1.12 (0.80-1.56)	1.58 (1.15-2.17)	0.004
Model II	1.00	1.59 (1.13-2.24)	1.88 (1.26-2.80)	<0.001	1.00	1.14 (0.82-1.60)	1.65 (1.20-2.27)	0.002
Model III	1.00	1.69 (1.14-2.51)	1.66 (1.00-2.75)	0.006	1.00	1.02 (0.70-1.49)	1.49 (1.02-2.16)	0.03
**IBS-D**									
Crude	1.00	1.11 (0.71-1.74)	0.98 (0.54-1.77)	0.86	1.00	1.27 (0.85-1.89)	1.17 (0.77-1.75)	0.46
Model I	1.00	1.10 (0.70-1.72)	0.95 (0.53-1.73)	0.94	1.00	1.28 (0.85-1.90)	1.17 (0.78-1.76)	0.44
Model II	1.00	1.12 (0.71-1.75)	0.99 (0.55-1.79)	0.83	1.00	1.29 (0.86-1.93)	1.20 (0.79-1.81)	0.38
Model III	1.00	1.34 (0.81-2.22)	1.16 (0.59-2.30)	0.37	1.00	1.43 (0.90-2.28)	1.21 (0.74-1.98)	0.46
**IBS-M**									
Crude	1.00	1.05 (0.64-1.73)	0.80 (0.40-1.62)	0.69	1.00	1.05 (0.68-1.62)	1.06 (0.68-1.63)	0.78
Model I	1.00	1.04 (0.63-1.71)	0.78 (0.38-1.56)	0.61	1.00	1.05 (0.68-1.62)	1.06 (0.69-1.64)	0.77
Model II	1.00	1.09 (0.66-1.79)	0.83 (0.41-1.68)	0.79	1.00	1.07 (0.69-1.66)	1.10 (0.70-1.70)	0.67
Model III	1.00	1.25 (0.72-2.19)	1.14 (0.54-2.42)	0.50	1.00	1.16 (0.70-1.94)	1.38 (0.83-2.30)	0.20
**IBS-U**									
Crude	1.00	0.74 (0.48-1.15)	0.92 (0.56-1.53)	0.40	1.00	1.17 (0.83-1.66)	1.13 (0.80-1.61)	0.48
Model I	1.00	0.73 (0.47-1.12)	0.88 (0.53-1.46)	0.30	1.00	1.18 (0.84-1.67)	1.14 (0.80-1.62)	0.44
Model II	1.00	0.77 (0.49-1.18)	0.96 (0.58-1.60)	0.51	1.00	1.21 (0.86-1.72)	1.20 (0.84-1.71)	0.31
Model III	1.00	0.78 (0.47-1.31)	1.25 (0.70-2.23)	0.81	1.00	1.36 (0.90-2.05)	1.31 (0.85-2.01)	0.21

a *Values are OR (95% CIs)*.

*Model I: adjusted for supplement use*.

*Model II: adjusted for dietary fiber intake*.

*Model III: adjusted for age, sex, energy, BMI, physical activity, smoking status, medication use, disease history (diabetes, colitis), psychological distress, regular meal pattern, chewing sufficiency, fluid consumption, breakfast skipping, dental status, and dietary fiber intake*.

## Discussion

In this cross-sectional study, we found that coffee and caffeine intake was associated with a greater odds of IBS in the whole study population. The association between caffeine and odds of IBS was also significantly positive among women and overweight or obese subjects (BMI ≥ 25 kg/m^2^). In addition, we found a significant relationship between caffeine intake and severity of IBS symptoms among overweight or obese subjects (BMI ≥ 25 kg/m^2^).

We found that coffee and caffeine consumption was associated with a greater odds of IBS. In line with our findings, several other studies have also indicated that coffee consumption was associated with symptoms of IBS (11, 21, 22). In a study in the Netherlands, coffee intake by IBS patients was not associated with functional bowel symptoms (23). However, the prevalence of IBS in general Dutch adult population appears to be lower than that in other countries. Several mechanisms may explain the significant positive association between coffee and caffeine intake and odds of IBS. Impaired gastrointestinal neural control due to dysregulations in hypothalamic-anterior pituitary-adrenocortical axis (HPA) and increased secretion of stress hormones has been implicated in the pathophysiology of IBS (24). Some studies have suggested that coffee may activate the HPA axis and elevate the stress hormones including cortisol, epinephrine, and norepinephrine (25, 26). Therefore, coffee consumption may increase the chance of IBS by altering HPA function. In addition, both coffee and caffeine stimulate gastric acid secretion and this may irritate the intestine and might lead to injury of the intestinal tissue (27, 28). Finally, caffeine blocks binding of gamma aminobutyric acid (GABA) to GABA receptors (29) and lack of GABA's effect help increasing the irritability and hyperactivity of the intestine (30).

In our study, we found a gender difference in the association between caffeine intake and odds of IBS; such that caffeine intake was linked to a greater chance of IBS in women, but not in men. The slower metabolism of caffeine among women than that in men might be a possible explanation for this gender discrepancy (31). In addition, we observed a significant association between caffeine intake and odds of IBS among overweight or obese (BMI ≥ 25 kg/m^2^), but not in normal weight subjects (BMI <25 kg/m^2^). Previous studies showed that the metabolism of caffeine is slower in overweight or obese people compared to that in normal-weight individuals (32). This may explain, at least in part, the positive association between caffeine consumption and odds of IBS in this population.

We found a significant relationship between caffeine intake and severity of IBS symptoms among overweight or obese subjects (BMI ≥ 25 kg/m^2^). However, we failed to find any significant association between coffee consumption and odds of IBS severity. It must be noted that number of people with IBS in this study was 748 subjects, most of them had mild to moderate IBS symptoms. Therefore, this might partly justify this finding. In terms of IBS subtypes, we found that coffee and caffeine intake was associated with increased chance of IBS-C. One explanation for this observation might be that the diuretic effect of caffeine can in turn lead to dehydration (33) and finally constipation. Theophylline, a chemical in tea, causes extracellular dehydration presumably *via* increase the renal glomerular filtration rate and decrease tubular reabsorption. Theophylline-induced dehydration increases water extraction from the stools and as a result, it worsens constipation (34). Decreased absorption of magnesium following coffee consumption may be another explanation for this finding (35). Laxative effect of magnesium is important for maintaining bowel regularity (36). Cholorogenic acid, another compound in coffee, is poorly absorbed and may be effective in creating looser stools by increasing the osmotic pressure in the intestine (37). In our study, the amount of chlorogenic acid in coffee was not measured. However, coffee content of chlorogenic acid should be taken into account when interpreting our findings.

This study has several strengths. Being the first study examining the association of coffee and caffeine intake and odds of IBS in general population, large sample size of the study, and considering the wide range of potential confounders in statistical analyses are among the strengths of the study. However, some limitations must be considered. The cross-sectional design of the study does not allow us to confer casual relationships between coffee and caffeine intake and IBS. In this study, we used self-reported questionnaires to assess the exposure and outcome. Therefore, misclassification of study participants in terms of exposure and outcome might be occurred.

In conclusion, the present study showed a significant positive association between coffee and caffeine intake and odds of IBS in the whole population. In addition, we found a significant positive association between caffeine intake and odds of IBS among women and subjects with BMI ≥ 25 kg/m^2^.

## Data Availability Statement

The raw data supporting the conclusions of this article will be made available by the authors, without undue reservation.

## Ethics Statement

The study protocol was ethically approved by the Regional Bioethics Committee of Isfahan University of Medical Sciences.

## Author Contributions

GK and AS-M contributed in conception, design, research, statistical analyses, data interpretation, and manuscript drafting. AHK and PA contributed in conception, design, and data interpretation. AE supervised the study. All authors contributed to the article and approved the submitted version and final manuscript for submission.

## Conflict of Interest

The authors declare that the research was conducted in the absence of any commercial or financial relationships that could be construed as a potential conflict of interest.
